# The Ward Ceiling, the Roof and Walls

**Published:** 1912-06-15

**Authors:** 


### THE MAKING OF A MODERN HOSPITAL.*

### The Ward Ceiling, the Roof and Walls.

The absolutely flat ceiling formerly used for wards has now been replaced in most modern institutions by a slightly concave or rounded ceiling. The essentials of a good ceiling are that it should be strong, smooth, without angles or superfluous concavities, projections, or ornamentation. Some stress has been laid by most authorities on the necessity of avoiding all woodwork in the ceiling; in newer hospitals the rule is that the whole ward should be made, as far as possible, of non-inflammable, hard material. Lightness and durability are necessary in the ceiling. Where ceiling ventilation is used care should be taken that the ventilators are readily accessible for cleaning, and repair. At the Johns Hopkins Hospital a somewhat elaborate plan of ceiling ventilation is used; a similar method has been employed at the Nuremberg Hospital and at the Military Hospital at Brussels. On the whole, however, such methods are not desirable. The old cross-beam ceiling ventilation (a good example is furnished by the wards in the medical (Hunt's) building at Guy's Hospital) is said to be good, but experience shows that the air in the wards where it is used is never up to the standard of purity exacted in modern hospitals. Ceiling lighting is quite undesirable. There is no need for it if the window space of the ward is in accordance with modern requirements, and it introduces several disadvantages. It is still used sometimes in the annexes. A particularly objectionable custom is the use of ceiling or roof windows in the bathroom and lavatories.

The ceilings are best painted in the same manner as the walls (see below), so as to harmonise with the general colour scheme of the ward. In the newer hospitals, where excessive height of the walls has been guarded against, the ceiling is within easy reach and can readily be cleaned without disturbing the occupants of the beds.

### Tiles or Slates for Roofing?

It is highly desirable that the hospital should possess a first-class roof, and institutional architects would do well to pay more attention than has hitherto been done to this very important point. Such questions as temperature, impermeability to moisture, durability of material, and appearance ought to be taken into consideration.

Among the materials now most commonly used for the roofing of hospitals are the following:

1. Tiles.—These are either: (*a*) Plain baked tiles, made of clay; (*b*) ribbed baked tiles; (*c*) metallic tiles; and (*d*) composition tiles, made of one or other of the compositions described below.

The use of tiles is generally recommended for suburban and rural hospitals. Tiles have also been employed with great success in the construction of the roofs of several of the new hospitals. Depage and Vandervelde consider that they give "a rustic appearance" to the buildings which is undesirable in an urban building. A better objection to their use is that they form roofs which permit the passage of heat in greater degree than slate does. On the other hand they are economical, clean, easily fixed, easily replaced when injured, and from a point of view of appearance generally satisfactory. It is stated that tiled roofs harbour more rats than any other form of roof, but this does not appear to be of any institutional importance! Various kinds of tiles are on the market. The requirements for a good tile are that it should be light, elastic to a certain extent so as not to be too brittle (the ribbed tiles are more elastic than the smooth), and not too porous. Most tiles, even when glazed or semi-glazed (in which form they are more expensive), absorb a certain amount of water which is readily evaporated by the heat, so that the air in tiled wards is more moist than in those with slate roofs. English-made tiles are preferable to imported varieties, and are much cheaper. A very excellent tile is that used by the Garden Tenants Co-partnership Association (Co-partnership Tenants, Limited): it is a ribbed tile made at Letchworth, elastic and light, and seems particularly suitable for institutional use. Composition, metallic, and "patent" tiles of various descriptions have been tried for hospital roofing with varying results. The great objection to their use is their almost prohibitive expense.

2. Slates.—Slate roofs are preferred by most modern authorities, and most of the new clinics are roofed with this form of material. The slate roof has a lower inclination than the tiled roof; it goes well with all forms of architecture, and adds greatly to the dignity and appearance of a large building, while it is equally suitable for small wards. It forms a heavy roof, which is impermeable, clean, and from the point of temperature quite satisfactory. Slate roofing, however, is expensive, and even when the artificial slate (which is less costly) is used, a slate roof is a dear necessity. Yet when everything is considered it is perhaps the most suitable form of roof material for a hospital that we possess at present. It is specially suitable for hospitals in warm climates, which are now, curiously enough, generally roofed with the much warmer tiles.

3. Metallic Sheets or Metal Plaques.—The most common form of this kind of roofing material is galvanised iron. When used it is laid on close boarding and lined with felt. For a tropical or emergency hospital it is a good form of roof material, but its use is not permissible in a modern hospital, except for temporary roofing purposes. Metallic plaques, which are used in the same way as tiles or slates, are far too expensive for hospital use and offer no advantages over the cheaper forms of roofing material. A lead roof is expensive and objectionable.

* Previous articles appeared on Oct. 7, 14, 21, 28, Nov. 4, 18, Dec. 9, 30, Feb. 10, March 16, April 6, 20, May 4, 18, June 1

4. COMPOSITION. — This material, of which several varieties compete for popularity, has been tried in Switzerland, America, and Germany, and more recently in Russia, for hospital use. The results are said to be excellent; the best example of a composite roof is found in the wood-cement (*Holzcement*) roof of some of the wards at Eppendorf. Wood-cement is a composition of sawdust and cement, which is subjected to great pressure, so that it forms a homogeneous mass from which large tiles are cut. These tiles are further impregnated with a tar composition. When completed they are strong, elastic, and absolutely watertight. They are laid down like slates, and the joints are strengthened with tar composition so that the whole roof is impermeable. As a further precaution a thin layer of earth is spread over the roof, on which grass is permitted to grow, which is said further to strengthen the roof. At Eppendorf this material has been found excellent, and it is well worth a trial in English hospitals, as it is relatively cheap. A few Continental hospitals, especially French ones, have lately tried a composition in which volcanic tufa is mixed with a light cement. This composition is much heavier than wood-cement and much less elastic, but is said to be stronger and to wear better. The wearing qualities of wood-cement are, however, to judge by the roofs of the Eppendorf wards, so good that no one is likely to find fault with them.

Compositions in which cork, india-rubber, various forms of bitumen, and iron wire are incorporated are generally too expensive to justify a trial.

Finally there is the glass roof, the best form of which is that in which the glass is protected by zinc network. This is only suitable for certain annexes of the special departments, and its costliness prevents it from being much used. Attic rooms or a large amount of free space between roof and ceiling should be avoided; high roofs are undesirable in every way, as they increase the expenses of construction without bringing compensatory advantages.

### WALL MATERIALS.

In a former article we briefly outlined the essentials of the ward wall. These differ in no way from those of a first-class dwelling-house, but it is well that the architect should bear in mind that minor faults, which may not give rise to much inconvenience in a private house, are often a source of great worry in a hospital. Wooden walls are best eliminated. It is true that they have often been used with conspicuous success, and where cheapness with excellence is desired a hospital can hardly do better than build its wards on the Moabite system, in which the pavilions are simple wooden structures lined inside with brick. Even the Moabite Hospital, however, has found it preferable, in its new pavilions, to depart from its own model and to use brick only for the walls; this slightly increases the expense, but obviates the serious objections which can be brought against a wooden wall—the chief of which is its want of durability, since a wooden structure stands in constant need of repair. Wooden walls are also liable to attract moisture. In damp and hot climates this is a matter of great importance, and most tropical hospitals possess several damp interceptors in each wall; these damp-proof courses are usually made of tarred brick laid in cement. Moisture rises in walls from the soil, owing to capillary attraction, to a height of 32 feet above ground level.

The best material for walls is undoubtedly brick. "We much prefer these to stone walls," remark Depage and Vandervelde in their recent treatise on hospital construction, "since the latter slowly disintegrate and always present a degree of humidity which it seems impossible to eliminate." The same authors recommend the Knapen system for hospital use. This system has been used at the Louvre and in certain public buildings, but has so far not been utilised in hospital construction. A full description is contained in Knapen's book "Hygrométrie du bâtiment," to which readers must be referred for further details. The system is based on a dual principle: extraction of the wall moisture, in the form of water or water vapour, by continuous automatic atmospheric syphon-suction, and the aeration of the wall itself.

The ward wall should be moderately thick, for it must shut out not only cold and damp and heat, but also noise. The interposition of aluminium plating has been tried in America in private rooms; such interposition is stated to eliminate all external noise and make the room sound-proof. Double walls with air, cork, or charcoal spaces between have also been tried; these certainly deaden the sound and are a good protection against cold, enabling the temperature of the ward to be kept equable in both winter and summer, but they are expensive and not popular. Plain brick walls, with the bricks well laid, the outside cemented or rough-casted, and the inside plastered and painted, are now generally favoured. The wall covering is a matter of considerable importance. It should prevent wall absorption. The foul odour that is noticeable in many of the wards of older hospitals, notwithstanding the fact that these wards are not overcrowded and are hygienically managed, is due to the absorption of such organic material by the walls which, when the temperature is raised, give off water vapour loaded with these impurities. The best way to overcome this difficulty of preventing absorption is to coat the surface of the wall with an impervious, non-absorbent enamel. Many varieties of such enamels are on the market; their merits vary much. In Belgium stucco has been much used as a wall covering for hospitals; it must be highly polished, and frequently well washed. A better wall covering is a close grain cement that can be painted with a washable oil paint. More expensive is a tiled surface, the tiles being of coloured enamel, with a mosaic dado carried up the lower part of the wall from the floor. Wall connections, such as electric-light wires, bell wires, switches, etc., must be laid in tubes flush with the wall surface wherever possible.

